# Maternally-Activated Lineage Tracing ( *Raeppli* ) To Determine Anlagen Size in *Drosophila*

**DOI:** 10.17912/micropub.biology.001185

**Published:** 2024-08-28

**Authors:** Michell Goyal, Keith Maggert

**Affiliations:** 1 University of Arizona, Tucson, Arizona, United States; 2 Molecular and Cellular Biology, University of Arizona, Tucson, Arizona, United States

## Abstract

We used *Raeppli* , a sophisticated fluorescent lineage tracing system developed for *Drosophila* , to map cell clones beginning at the earliest possible stage in development. By expression of the ϕC31 Integrase (the final step in activating lineage marking) in nurse cells and oocytes, we reduced the methodological and biological variation in cell lineage analysis. We characterized the number of cells in fully-developed larval salivary glands, and thereby inferred the number of cells in embryonic anlage, the number of divisions during differentiation, and the variation in clonal behavior. This approach – novel for *Raeppli* – represents a powerful addition to lineage analysis in *Drosophila* .

**Figure 1. Maternally-Activated Raeppli:**
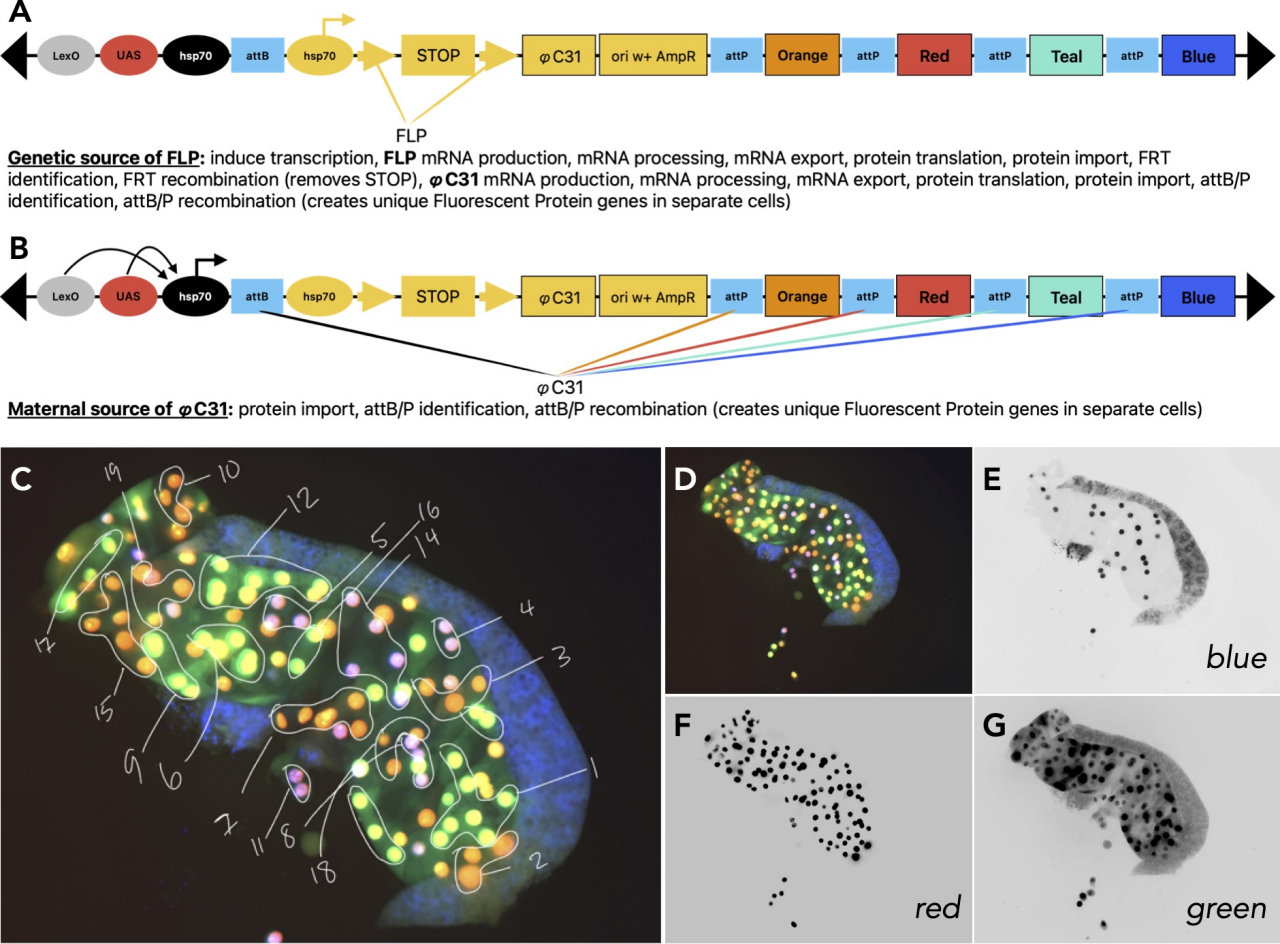
**(A)** Schematic of the *Raeppli* live fluorescence lineage marking system, as developed by Kanca and colleagues (Kanca *et al.* 2014). Activation begins with expression of the FLP Recombinase and takes many steps until a cell lineage is marked by a fluorescent protein gene. **(B)** Maternal supply of the Integrase (ϕC31) expedites cell lineage marking, removing much of the variation and producing the minimum number of clones possible for an organism. **(C)** *Raeppli* expression in a whole mount salivary gland using maternally supplied Integrase, visualized using epifluorescence microscopy. The blue tissue surrounding the salivary gland corresponds to fat body tissue, which was not analyzed. **(D-G)** Same merged image as (C) without annotation, and separated into each fluorescent channel (as indicated), inverted, and adjusted for bright and contrast.

## Description

We sought to trace *Drosophila* cells from early embryogenesis to mature larval tissues, in an unbiased fashion and with as great a robustness against differential number of subsequent lineage-specific cell divisions as possible. *Raeppli* , developed for *Drosophila* , is a powerful and adaptable lineage tracing system for mapping cell clones by inducing a genome rearrangement to create one of four fluorescent protein genes which express in the cell in which the rearrangement occurs (Kanca et al. 2014) . The *Raeppli* transgene contains two *FLP* *Recombination* *Target* sequences ( *FRT* s) that flank a transcriptional Stop cassette such that tissue-specific or inducible ( *e.g.* , heat shock) FLP Recombinase expression causes excision of the Stop cassette, activating expression of viral (ϕC31) Integrase. Integrase then catalyzes one of four possible site-specific exchanges within the transgene to create LexA- or GAL4-driven expression of one of four possible fluorescent proteins — E2-Orange (orange), mKate (red), TFP (teal), or TagBFP (blue) ( **Figure 1A** ). Cells in which the rearrangement occurs, and in all cells in subsequent lineages, are labeled by fluorescence. Given these four rearrangements, plus the opportunity to use *Raeppli* as a homozygote (or two or more *Raeppli* cassettes in non-allelic locations), over a dozen possible fluorescent label combinations can be discriminated.

Normally, FLP and heat-shock induced gene expression is not effective in early embryogenesis, limited by the very short ( *ca.* 9 minutes) S-Phase time, chromatin structure, and other characteristics of the Mid-Blastula Transition (MBT, also Zygotic Genome Activation, ZGA, and “onset of zygotic transcription”) (Yuan et al. 2016) . Reliable synchronized early activation of *Raeppli* is limited by the heterogeneity of development: different genotypes exhibit different timing for embryogenesis events, and even genetically identical organisms can express stochastic differences ( *i.e.* , “developmental noise”). Further heterogeneity arises from unsynchronized ovulation, which is simultaneous to activation of embryonic development (Loppin et al. 2015) . Together these factors result in a broad distribution of the timing of *Raeppli* activation, which could seriously impact accurate estimation of cell counts in mature organs or tissues. We therefore desired to induce *Raeppli* as early as possible, and at a uniform developmental stage, regardless of intra-individual variation or differences in times of egg laying in large populations from which eggs/embryos are collected.

We used a maternally-expressed ϕC31 Integrase (driven by the *nanos* promoter, active in oogenesis) to create eggs that contain fully-active Integrase that *Raeppli* -bearing sperm could fertilize. As soon as the paternally-packaged *Raeppli* chromosomes are “unpackaged” and subject to transcription, the waiting Integrase can rearrange and activate the *Raeppli* transgene ( **Figure 1B** ), obviating the need for FLP Recombinase (and all of the delays and heterogeneity arising from transcription, mRNA processing, export, translation, and nuclear import of FLP Recombinase and Integrase, plus genome rearrangement by FLP Recombinase).

We created embryos by crossing females expressing maternal Integrase and possessing a ubiquitously-expressed GAL4 to males containing the *Raeppli* transgene. F1 larvae were identified and dissected for analysis using fluorescence microscopy. We validated this use of maternally-activated *Raeppli* by determining the number of cells in the anlage of the salivary glands by analysis of the mature 3rd instar larval salivary glands. The anlage are identified by gene expression by stage 9, as a visible placode by stage 10, and begin to invaginate by stage 11 (Maruyama and Andrew 2011) . Salivary glands are determined by the overlap between *Sex combs reduced* , *Abdominal-B* , *forkhead* , and *teashirt* , use *decapentaplegic* and *EGF* for early subdivision and differentiation, and can be identified by *dCREB-A* expression (Andrew et al. 2000) . It is important to note that none of these genes is restricted to the salivary glade anlage, and so none can be used easily as a means to identify salivary gland primordial cells. It has been reported that 100 cells are in each bilateral anlagen (Maruyama and Andrew 2011) , but it is not known if these 100 are daughters of a smaller subset of determined cells, or if all of the 100 cells (and their descendants) are represented in the mature tissue.

We analyzed a dozen intact (paired, branched) third instar larval salivary glands, grouping cells with identical fluorescence characteristics and whose location was contiguous with cells exhibiting similar fluorescence, with the assumption that they arose from a common precursor at the MBT (stage 4). An example of the tissue is shown in Figure 1C and 1D, which exemplifies the type of data and the way we analyzed them. From these analyses, we made three salient observations.

First, as expected, despite the presence of Integrase in the egg at the time of fertilization, Integrase-mediated rearrangement did not occur until well into development. We infer this because if rearrangement of *Raeppli* occurred at the time of fertilization, male pronuclear chromatin remodeling, or even within the first few syncytial divisions, we expected that the entire salivary gland, or at least each lobe, would be uniform in fluorescence (Goyal 2023) . At the MBT, there are approximately 1000-2000 nuclei, and we infer that each nucleus in the syncytium independently underwent rearrangement. Although it is no surprise that expression of the genes in *Raeppli* are not expressed until the MBT (Schulz et al. 2015, Blythe et al. 2016) , it was a surprise that even the genome rearrangements (caused by Integrase) could also delayed until the MBT. We have no direct evidence that the rearrangement occurred at the MBT, but it seems both plausible and consistent with the number of clones we analyzed later in development. Such *en masse* rearrangement likely reflects a change in global chromatin architecture at the MBT, affecting not only transcription, but also site-specific recombination.

Second, there were on average 18 clones in each bilateral gland, indicating that each anlagen contained this number of cells. Across the dozen salivary glands we analyzed, there was a remarkable consistency in determination of anlagen cell number. The standard deviation was 4.3, sample size = 16. This is true between individuals and between lobes of the bilateral salivary gland. This consistency speaks to the robustness of both the methodological (induction time, cell labeling, *Raeppli* rearrangement) and biological (number of cells in anlagen) variation.

Third, labeled clones each contained primarily the same number of cells (N = 3.6 ±1.9, sample size = 127). There were a small subset that contained more than this number of cells (≥11), however we could not rule out that these were merely adjacent clones with the same fluorescent characteristics stemming from the same *Raeppli* rearrangement in multiple adjacent progenitor cells in the anlagen. This observation indicates that each cell in the anlagen divides the same number of times in salivary gland development. Further, we noted that clone arrangement (within the mature gland) exhibited no discernible pattern, indicating that there does not appear to be any subpopulations at the time of anlagen formation that lead to distinct subpopulations in the glands.

In terms of our original point about salivary gland development, it would seem there is a discordance between the reported anlagen size (100 cells) and the final number in the mature glands (18 ± 4.3 clones x 3.6 ± 1.9 cells per clone = 64.8 ± 37.5; t-test indicates the ability to reject the null hypothesis; *t* -statistic = 3.755, *N* = 16, *df* = 14, *H*_0_ states that 100 cells is drawn from the population of 64.8 ± 37.5 cells).

Although one may argue that similar information can be obtained by confocal microscopy coupled with immunofluorescence or marker gene expression, challenges exist with those approaches that make this use of *Raeppli* a valid addition to the tools available for analyzing developmental processes. First, confocal microscopy and 3D reconstruction is both costly and data- and time-intensive. Maternally-activated *Raeppli* makes easy lineage analysis available for a low cost, and within a single image. Second, not all samples are amenable to confocal analysis. With *Raeppli* , tissues can be analyzed live or fixed, as whole-mount or dissected. This reduces the possibility that cells may be missed and allows for subsequent protocols that are not compatible with confocal ( *e.g.* , fluorescent *in situ* hybridization, immunofluorescence). Third, other forms of live fate mapping ( *e.g.* , G-TRACE (Evans et al. 2009) ) require an appropriate cell marker, which is not always available. Further, not every cell that contributes to a tissue may express a marker to the level that it is reliably and unequivocally identifiable. Maternally-activated *Raeppli* circumvents these problems which, in some cases, may limit or confound experimental approaches.

We also note that the development of *Raeppli* fluorescent proteins that are nuclear-localized (as we have used above) and that are membrane bound (by dint of C-terminal prenylation, Kanca et al. 2014), plus the panoply of ubiquitously-expressed (or even tissue-specific) GAL4, provide significant flexibility to the approach we describe. **(A)** Schematic of the *Raeppli* live fluorescence lineage marking system, as developed by Kanca and colleagues (Kanca et al *.* 2014). Activation begins with expression of the FLP Recombinase and takes many steps until a cell lineage is marked by a fluorescent protein gene. **(B)** Maternal supply of the Integrase (ϕC31) expedites cell lineage marking, removing much of the variation and producing the minimum number of clones possible for an organism. **(C)** *Raeppli* expression in a whole mount salivary gland using maternally supplied Integrase, visualized using epifluorescence microscopy. The blue tissue surrounding the salivary gland corresponds to fat body tissue, which was not analyzed. **(D-G)** Same merged image as (C) without annotation, and separated into each fluorescent channel (as indicated), inverted, and adjusted for bright and contrast.

## Methods

Flies were of genotype *y* ^1^ *v* ^1^ P{ *y* ^+t7.7^ =nos-phiC31\int.NLS} *X/w* ^*^ ; P{ *w* ^+mC^ =Raeppli-NLS} *28A* /+; P{w ^+mC^ =Act5C-GAL4} *17bFO1* /+, the F1 progeny of *y* ^1^ *v* ^1^ P{ *y* ^+t7.7^ =nos-phiC31\int.NLS} *X* ; +; P{ *w* ^+mC^ =Act5C-GAL4} *17bFO1* / *TM6B* , *Tb* ^1^ mothers and *w* ^*^ ; P{ *w* ^+mC^ =Raeppli-NLS} *28A* fathers. Flies were mated and allowed to lay eggs for 3-4 days. Adults were removed and the embryos and larvae were allowed to age until reaching wandering third instar stage. Larvae were dissected using 5S forceps in 1X PBS. Salivary glands were placed in 20 µL PBS (with or without 10 ng/mL Hoescht-33342) on glass slides, and covered with a glass coverslip without squashing. Salivary glands were visualized on an AxioZoom.v16. Images were taken on an AxioCam using Zeiss filter set series 00 (excitation 530-585, split 600, emission 615), 38HE (excitation 470/40, split 495, emission 525/50), 46 (excitation 500/20, split 515, emission 535/30), 49 (excitation 365, split 395, emission 445/450).

## Reagents

*Drosophila* strain genotypes (and their respective Bloomington *Drosophila* Stock Center numbers, at https://bdsc.indiana.edu as of March 1, 2024):

*X* -linked Raeppli: P{ *w* ^+mC^ =Raeppli-NLS} *3C* , *y* ^1^ *w* ^*^ ; +; +; + (Stock #55085)

Chromosome *2* -linked Raeppli: *w* ^*^ ; P{ *w* ^+mC^ =Raeppli-NLS} *28A* ; +; + (Stock #55086)

Chromosome *3* -linked Raeppli: *w* ^*^ ; +; P{ *w* ^+mC^ =Raeppli-NLS} *89A* ; + (Stock #55088)

Maternal Integrase source: *y* ^1^ *v* ^1^ P{ *y* ^+t7.7^ =nos-phiC31\int.NLS} *X* ; +; +; + (extracted from Stock #25709)

Maternal Integrase source: *y* ^1^ *sc* ^1^ *v* ^1^ P{ *y* ^+t7.7^ =nos-phiC31\int.NLS} *X* ; +; +; + (extracted from Stock #25710)

Ubiquitous GAL4 source: *y* ^1^ *w* ^*^ ; +; P{ *w* ^+mC^ =Act5C-GAL4} *17bFO1* / *TM6B* , *Tb* ^1^ ; + (Stock #3954)

We obtained the three nuclear *Raeppli* transgenes from the Bloomington *Drosophila* Stock Center and created a genotype that contains all three (P{ *w* ^+mC^ =Raeppli-NLS} *3C* , *y* ^1^ *w* ^*^ ; P{ *w* ^+mC^ =Raeppli-NLS} *28A* ; P{ *w* ^+mC^ =Raeppli-NLS} *89A* ). This strain can be obtained from us directly by request. Males of this genotype can be crossed with females of any number of *Drosophila* strains expressing maternally-supplied Integrase ( *e.g.* , *y* ^1^ *v* ^1^ P{ *y* ^+t7.7^ =nos-phiC31\int.NLS} *X* , *y* ^1^ *sc* ^1^ *v* ^1^ P{ *y* ^+t7.7^ =nos-phiC31\int.NLS} *X* ), and the female offspring (which will contain three *Raeppli* transgenes) or the male offspring (which will contain two) analyzed.

## References

[R1] Andrew DJ, Henderson KD, Seshaiah P (2000). Salivary gland development in Drosophila melanogaster.. Mech Dev.

[R2] Blythe SA, Wieschaus EF (2016). Establishment and maintenance of heritable chromatin structure during early Drosophila embryogenesis.. Elife.

[R3] Evans CJ, Olson JM, Ngo KT, Kim E, Lee NE, Kuoy E, Patananan AN, Sitz D, Tran P, Do MT, Yackle K, Cespedes A, Hartenstein V, Call GB, Banerjee U (2009). G-TRACE: rapid Gal4-based cell lineage analysis in Drosophila.. Nat Methods.

[R4] Goyal M. 2023. Gonomery meets anlagen: On the etiology of bilateral gynandromorphy in Insecta [dissertation]. [Tucson (AZ)]: University of Arizona.

[R5] Kanca O, Caussinus E, Denes AS, Percival-Smith A, Affolter M (2013). Raeppli: a whole-tissue labeling tool for live imaging of Drosophila development.. Development.

[R6] Loppin B, Dubruille R, Horard B (2015). The intimate genetics of Drosophila fertilization.. Open Biol.

[R7] Maruyama R, Andrew DJ (2011). Drosophila as a model for epithelial tube formation.. Dev Dyn.

[R8] Schulz KN, Bondra ER, Moshe A, Villalta JE, Lieb JD, Kaplan T, McKay DJ, Harrison MM (2015). Zelda is differentially required for chromatin accessibility, transcription factor binding, and gene expression in the early Drosophila embryo.. Genome Res.

[R9] Yuan K, Seller CA, Shermoen AW, O'Farrell PH (2016). Timing the Drosophila Mid-Blastula Transition: A Cell Cycle-Centered View.. Trends Genet.

